# Dialog between Kingdoms: Enemies, Allies and Peptide Phytohormones

**DOI:** 10.3390/plants10112243

**Published:** 2021-10-21

**Authors:** Irina Dodueva, Maria Lebedeva, Lyudmila Lutova

**Affiliations:** Department of Genetics and Biotechnology, Saint Petersburg State University, Universitetskaya Emb. 7/9, 199034 Saint Petersburg, Russia; m.a.lebedeva@spbu.ru (M.L.); l.lutova@spbu.ru (L.L.)

**Keywords:** plant–microbe interaction, plant parasitic nematodes, effectors, CLAVATA3/EMBRYO SURROUNDING REGION-RELATED (CLE), PLANT PEPTIDES CONTAINING SULFATED TYROSINE (PSY), phytosulphokines (PSK), C-TERMINALLY ENCODED PEPTIDES (CEP), INFLORESCENCE DEFICIENT IN ABSCISSION (IDA), RAPID ALKALINIZATION FACTOR (RALF), PLANT ELICITOR PEPTIDES (PEP)

## Abstract

Various plant hormones can integrate developmental and environmental responses, acting in a complex network, which allows plants to adjust their developmental processes to changing environments. In particular, plant peptide hormones regulate various aspects of plant growth and development as well as the response to environmental stress and the interaction of plants with their pathogens and symbionts. Various plant-interacting organisms, e.g., bacterial and fungal pathogens, plant-parasitic nematodes, as well as symbiotic and plant-beneficial bacteria and fungi, are able to manipulate phytohormonal level and/or signaling in the host plant in order to overcome plant immunity and to create the habitat and food source inside the plant body. The most striking example of such phytohormonal mimicry is the ability of certain plant pathogens and symbionts to produce peptide phytohormones of different classes. To date, in the genomes of plant-interacting bacteria, fungi, and nematodes, the genes encoding effectors which mimic seven classes of peptide phytohormones have been found. For some of these effectors, the interaction with plant receptors for peptide hormones and the effect on plant development and defense have been demonstrated. In this review, we focus on the currently described classes of peptide phytohormones found among the representatives of other kingdoms, as well as mechanisms of their action and possible evolutional origin.

## 1. Introduction

Land plants live in constant interaction with other organisms, predominantly microorganisms, which have different life strategies ranging from symbiosis to necrotrophic pathogenesis. Therefore, plants have to constantly negotiate with “enemies” (pathogens) and “allies” (symbionts, as well as beneficial epiphytes and endophytes). The colonization of a plant by various beneficial and harmful organisms is governed by a complex network system that has been developed in long co-evolution and which includes a molecular dialog between the interacting partners [1,2].

There is a constant signal exchange between the host plant and the organisms which colonize it. In particular, different effector proteins are produced by plant pathogens, secreted into the host plant tissues, which help to overcome plant defense system and/or to modulate host plant physiology [3,4]. The effector proteins also play an important role in the colonization of the plant by symbiotic organisms, e.g., mycorrhizal fungi [5] or nitrogen-fixing bacteria [6]. In its turn, a host plant can recognize the molecules secreted by a pathogen and activate the immune response by producing the proteins with antimicrobial activity and toxic compounds [1,7].

Phytohormones play an important role in the interaction of plants with pathogens and beneficial microbes. In response to pathogens, salicylic acid (SA), jasmonates (JA), and ethylene are the major plant “defense hormones”, whereas abscisic acid (ABA), auxins (mostly IAA), cytokinins, and brassinosteroids play a supportive role as modulators of plant defense systems [1,8]. The hormonal signaling network in plant immunity is complex and multi-level, and it includes a lot of feedback loops such as antagonism of JA- and SA-mediated immunity, which plays an important role in plant protection against pathogens with different lifestyle strategies [9,10]. JA and ethylene are also involved in the plant relationship with at least some beneficial organisms; for example, these hormones were shown to be accumulated at the early stages of plant infection by mycorrhizal fungi as well as rhizobia [11].

It is well known that certain bacterial and fungal pathogens can modulate the phytohormonal signaling networks to overcome plant defense. The most known pathway is the suppression of SA-mediated immunity which is often exploited by biotrophic pathogenic bacteria. For example, certain effectors of *Pseudomonas syringae* trigger the degradation of JAZ proteins, which repress/inhibit the expression of JA-mediated genes, thereby activating JA signaling and repressing SA-mediated immunity [12,13]. Moreover, some pathogens are able to produce substances that mimic plant hormones. For example, the same *P. syringae* can produce coronatine, a structural and functional analog of jasmonyl-l-isoleucine, which can interact with the JA receptor and which activates downstream signaling cascade leading to the repression of SA-dependent plant defense response [14]. Among beneficial plant-interacting organisms, there are also examples of modulation of plant immunity via manipulation with the defense phytohormones. Certain mycorrhizal fungi were shown to modulate ethylene signaling to possess immune-suppressive function [15], and some plant growth-promoting bacteria and fungi can also modulate plant defense via SA-JA antagonism or suppression of ethylene signaling [16].

On the other hand, plant colonization strategies are often aimed to modify plant growth in order to supply nutrition or create a habitat for a pathogenic or symbiotic partner. One way to reach these goals is to change the balance of main “growth-regulating” hormones, IAA and cytokinins, in the body of the host plant. In its turn, the IAA-cytokinin imbalance in the plant host can be achieved due to the ability of the colonizing organism to synthesize and secrete these phytohormones, or due to the effectors that can alter the level of active IAA and/or cytokinins, or their transport in the plant body, or which can affect the transduction of phytohormonal signals. In addition to the change of plant tissues growth, manipulation with IAA and/or cytokinin levels or alteration of plant responses to them can potentially attenuate SA signaling via hormonal cross-talk mechanisms [7]. The most striking example of such manipulation of IAA/cytokinin balance is provided by various plant pathogens (bacteria, fungi, protists, nematodes, arthropods) which induce the neoplasia of host plant tissues (tumors or galls) to create the habitat niche for themselves (reviewed in [17]). The genes encoding enzymes for IAA and cytokinin biosynthesis were first identified in the virulent plasmid of plant pathogenic bacterium *Agrobacterium tumefaciens* that are capable of transferring part of this plasmid into the host plant genome, leading to the increase of IAA and cytokinin content and tumor induction [17,18]. To date, genes for IAA and/or cytokinin biosynthesis were found in a variety of bacteria and fungi, including pathogenic, symbiotic, and free-living species, and also in plant-parasitic nematodes (reviewed in: [19,20]). The example of an effector that affects IAA/cytokinin balance in the host plant is the 19C07 protein of gall-inducing sugarbeet nematode *Heterodera schachtii*, which interacts with an IAA influx transporter, LAX3, influencing the direction of IAA transport [21].

**Figure 1:**
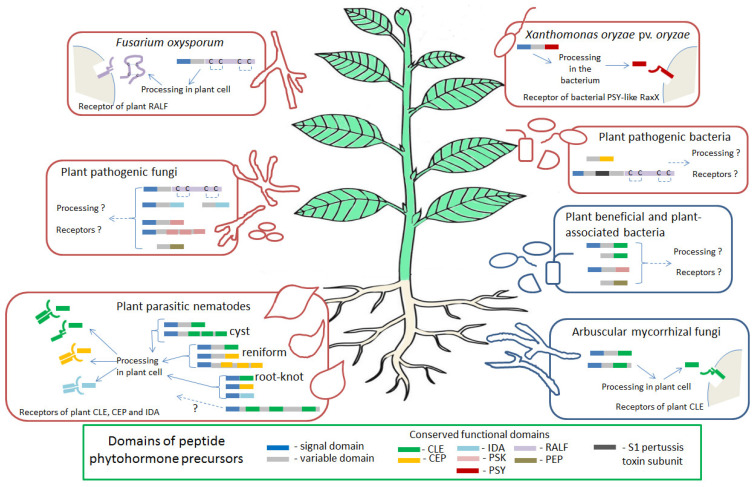
Peptide phytohormones of plant-interacting bacteria, fungi, and nematodes.

Our review focuses on a specific type of phytohormonal mimicry, based on the ability of certain plant pathogens and symbionts to produce the effector proteins, which mimic peptide hormones of a host plant (Figure 1). By now, about thirty families of peptide phytohormones have been identified in plants, and they can be divided into two types: secreted and non-secreted ones [22]. Secreted peptide phytohormones can be further divided into two large groups: post-translationally modified peptides and cysteine-rich peptides. Post-translationally modified peptides are usually synthesized as larger precursor proteins (hundreds of amino acids in length), which undergo subsequent post-translation modifications, including proteolytic processing, as well as hydroxylation and glycosylation of conserved proline and/or sulfation of tyrosine residues [23,24]. Cysteine-rich peptides contain 2–16 cysteine residues, which are necessary for the organization of their spatial structure through intramolecular disulfide bonds [25]. Precursor proteins of secreted peptide phytohormones usually include an N-terminal signal domain, which directs the peptide to the secretory pathway, a variable region, and the most functionally significant C-terminal domain, which is cleaved out from a precursor protein during proteolytic processing and which undergoes modifications of certain conserved amino acids residues [24,26]. Mature peptide phytohormones are then secreted to the apoplast, where they interact with the receptors on the plant cell surface. Receptors for most peptide phytohormones are serine-threonine protein kinases with an extracellular domain containing leucine-rich repeats (LRR-RLK, leucine-rich repeats containing receptor-like kinase), responsible for the interaction with peptide ligands [27,28]. Among the families of peptide phytohormones discussed in this review, CLAVATA3/EMBRYO SURROUNDING REGION-RELATED (CLE), PLANT PEPTIDES CONTAINING SULFATED TYROSINE (PSY), phytosulphokines (PSK), C-TERMINALLY ENCODED PEPTIDES (CEP), and INFLORESCENCE DEFICIENT IN ABSCISSION (IDA) are post-translationally modified peptides, whereas RAPID ALKALINIZATION FACTOR (RALF) peptides belong to cysteine-rich peptides and PLANT ELICITOR PEPTIDES (PEP)—to non-secreted peptides [26].

Various plant peptide hormones play a role in the defense from pathogens and herbivores and also in the interaction with beneficial microorganisms. Some families of peptide phytohormones have exclusively protective functions and are among the components of plant immunity, whereas other members of peptide phytohormones mostly play a role in the regulation of plant growth, but can be also involved in the defense response or plant–microbe interactions. The biosynthesis of the first discovered peptide phytohormone, a non-secreted peptide systemin, is induced by any mechanical damage of plant tissues, including injury by phytophagous insects [29,30]. The member of another family of non-secreted peptides, PEP, function as damage-associated molecular patterns (DAMPs): in *Arabidopsis*, mature PEP1 enters the extracellular space when the cell is destroyed due to the attack by pathogens or herbivores and activates local and systemic immune response [31]. Among the targets of PEP-induced immunity, there are genes encoding post-translationally modified PAMP-INDUCED SECRETED PEPTIDES (PIP), which also can be activated by the binding of pathogen-associated molecular patterns (PAMPs) at the plant cell surface as well as by SA treatment [32]. Among post-translationally modified peptide phytohormones, the components of plant immunity also include hydroxyprolinesystemins (HypSys), which are not homologous to systemin [33]. Among peptide phytohormones that participate in the defense response as well as in plant growth regulation, PSK, IDA, and PSY post-translationally modified peptides are known. Structurally related, the PSK and PSY peptides stimulate cell division and cell growth [34,35]; however, they also play a role in the protection from necrotrophic pathogens and response to tissue damage [36]. IDA peptides stimulate plant cell wall modifications, thereby increasing cell separation during leaf and flower abscission as well as lateral root emergence [37]. In addition, certain plant pathogens could manipulate the levels of IDA peptides to decrease plant defense. For example, the *IDL6* gene of *Arabidopsis* was shown to be upregulated upon attack by *P. syringae,* which leads to the increased expression of polygalacturonase-encoding genes resulting in the decrease in pectin content, which makes the leaf less resistant to the infection [38]. Other players of cell growth modification, RALF peptides and their receptor FERONIA (FER), also function as essential regulators of plant immunity: activated FER directly phosphorylates and destabilizes the MYC2 transcription factor, which is a master regulator of JA signaling [39].

In the scientific literature of recent years, there are data on the identification of peptide phytohormones of different classes outside the plant kingdom. To date, genes encoding precursor proteins of the CLE, CEP, RALF, IDA, PSK, PSY, and PEP peptide phytohormones have been identified in a variety of phytopathogens (bacteria, fungi, and nematodes) [40,41] (Table 1). Among plant-beneficial microorganisms, *CLE*-like genes were found in the genomes of some of mycorrhizal fungi [42], as well as plant-growth-promoting *Actinobacteria* species [41]. For some of these peptide phytohormones of non-plant origin, such as the CLE peptides of plant-parasitic nematodes [43], the mechanisms of secretion and processing, binding with plant receptors, as well as a role in the plant colonization and regulation of plant growth have been studied in details. At the same time, numerous new examples of such effectors, such as the PSK peptides of phytopathogenic bacteria and fungi, have been identified quite recently and are practically not studied at the moment [41].

The production of peptide phytohormones can be used by plant-colonizing organisms for different purposes. First, the effectors mimicking peptide phytohormones can help overcome the defense systems of host plants and increase the efficiency of colonization, as in the case of the PSY-like bacterial peptide RaxX [66]. Second, plant peptide hormone mimics can manipulate the growth of plant tissues to create the habitat and food source for the colonizer, as it was shown for the CLE and IDA peptides of plant-parasitic nematodes [43]. Likewise, the CLE peptides produced by arbuscular mycorrhizal fungi stimulate lateral root development thereby increasing mycorrhization of the host plant [42].

Thus, according to the recent data, peptide phytohormones are widespread outside the plant kingdom and can be used by organisms with different life strategies to interact with plants. Our review generalizes the data on peptide phytohormone synthesized by plant pathogens and symbionts, with a special focus on their mechanisms of action and possible evolutionary origin.

## 2. Peptide Phytohormones from Plant Pathogens: *Divide et impera*

Coevolution of plants and their pathogens is always a “race of arms”: there are well-known “gene-for-gene” relationships between pathogen virulence factors and host resistance genes that confer the capacity to recognize and to respond to very diverse PAMPs/DAMPs or effectors of pathogens. In turn, pathogens constantly evolve diverse secreted effectors to overcome plant immunity and to facilitate colonization [75]. Phytohormones also play an important role in the “war” between the plant and its invaders. SA, JA, and ethylene are necessary to prevent plant colonization by a pathogen, while IAA, cytokinins, ABA, gibberellins, brassinosteroids, and certain classes of peptide phytohormones play less pronounced roles in plant defense helping to restrict pathogen and redistribute resources within the plant [8].

However, some plant pathogens are able to use phytohormones for their own purposes, modulating the level of active phytohormones or the response to them in the host plant [14,19,20]. In particular, the ability to produce peptide phytohormones, which are used to change the growth of the host plant and to suppress its defense reactions, has become widespread among the phytopathogens from different kingdoms of the living world—bacteria, fungi, and animals (namely, nematodes) [40,43]. Below, we review examples of the peptide phytohormones found in plant pathogens in more detail.

### 2.1. Peptide Phytohormones of Plant Parasitic Nematodes

Plant-parasitic nematodes are widespread economically significant pests of different crops. According to phylogenetic data, it was supposed that plant parasitism has arisen at least four times independently in nematodes [76]. Plant-parasitic nematodes use a variety of feeding strategies: among them there are ecto- and endoparasites, which can be migratory or sedentary [77]. The typical life strategy of sedentary endoparasite plant-feeding nematodes is the formation of so-called feeding sites—highly metabolic large cells which provide nutrition for nematodes. Two groups of sedentary endoparasitic nematodes, the cyst nematodes (*Globodera* spp. and *Heterodera* spp.) and the root-knot nematodes (*Meloidogyne* spp.), also induce neoplasia of host root tissues—the formation of galls caused by increased division rate and rapid expansion of the cells close to the feeding site [78]. Cyst and root-knot nematodes have different strategies to create feeding sites. In the case of cyst nematodes, the fusion of hundreds of cells into a syncytium takes place, while root-knot nematodes induce repeated rounds of endoreduplication, nuclear division, and cell growth in the absence of cytokinesis, which results in giant cell formation. The formation of syncytium-type feeding sites is also typical for the group of the reniform nematodes (*Rotylenchulus* spp.)—semiendoparasites, which does not cause galling of the infected roots [43,77]. The feeding sites formation upon the infection with cyst, root-knot, or reniform nematodes is accompanied by the dramatic increase in the expression levels of core cell cycle genes [79,80].

The initial event of feeding site formation is the penetration of the stylet (mouth spear) of juvenile nematode into plant cells in the root cortex, endodermis, or pericycle. After that, the content of the esophageal glands of the nematode is injected into plant cells, followed by the expansion of a local group of cells. The secretion of these glands contains a variety of substances, which are responsible for nematode invasion, and also for the formation of feeding sites and galls. Among these substances, there are nematode-derived cytokinins, which are able to modulate plant cell cycle [81], as well as cell wall degrading enzymes, which are necessary to facilitate the nematode migration and the formation of feeding sites, as well as different types of effector proteins, including those which modify the direction of polar IAA transport and suppress plant resistance responses. In addition, the secretion of nematode esophageal glands contains proteins that mimic plant peptide hormones [77,78].

To date, the nematode-derived peptide phytohormones of the CLE, CEP, and IDA families have been identified [41,43]. The precursors of these peptides are secreted into the root cells after nematode penetration in the host plant, where they undergo processing in the plant body, and the resulting mature peptides are able to interact with plant receptors and activate downstream signaling pathway leading to growth response. The expression of nematode genes encoding peptide phytohormones is required for the successful invasion of the host plant by nematodes [59,64,82].

#### 2.1.1. CLE Peptides of Plant Parasitic Nematodes

Nematode *CLE* genes were first identified in the soybean cyst nematode *Heterodera glycines* [46,47], and later they were described in many other species of cyst, root-knot, and reniform nematodes [48,55,58,60,62,83]. The CLE precursor proteins encoded by nematode genes, like endogenous plant CLE precursors, include an N-terminal secretion signal domain and C-terminal CLE domain which is a functional part of all CLEs [49]. It is believed, that the N-terminal signal domain directs the CLE protein through the gland cell secretory pathway to package into secretory granules [43]. In addition, the CLE propeptides of all cyst and reniform nematodes include a variable domain (which is also typical for plant CLEs) and a so-called cryptic signal domain, which is located, as a rule, in the N-terminal portion of the variable domain and is necessary for trafficking the nematode CLEs to the apoplast by the host plant secretory pathway [50,62]. Some nematode species (as well as some plant species) have multidomain CLE peptides containing from two to nine CLE domains, which may have similar or different sequences [55,62]. Examples of such proteins are CLEs of potato cyst nematode *Globodera rostochiensis* [55] and so-called MAPs (*Meloidogyne* avirulence proteins) of *Meloidogyne* spp. [62].

As it was shown for multidomain CLEs of *G. rostochiensis* [56], nematode-derived CLE propeptides can undergo proteolytic processing and glycosylation by utilizing host plant cellular machinery to become bioactive CLE peptides. The resulting nematode-derived CLE peptides can mimic plant-derived CLEs via interacting with the LRR-RLK family receptors on the plant cell surface [48,51,52,53,56,57]. The dramatic increase in the *CLE* genes expression at the parasitic stages of nematode development [46,47,49,55], along with the fact that transgenic plants expressing double-stranded RNA complementary to nematode *CLEs* are resistant to nematode infection [82], indicate the important role of nematode-derived CLE peptides at least in the parasitism of cyst nematodes.

The cyst nematodes contain two groups of the *CLE* genes, one of which is related to plant genes encoding the A-type CLEs, and the other one—with the B-type CLE peptides. In *Arabidopsis* and other plants, functional analysis of CLE peptides divided them into two types, named A-type and B-type. A-type CLEs, which include CLV3, promote cell differentiation in the shoot and root apical meristems, whereas B-type CLEs act in the lateral meristems, procambium, and cambium, where they promote cell proliferation and inhibit cell differentiation into xylem elements [84]. The main targets of plant CLEs are the *WUSCHEL-related HOMEOBOX* (*WOX*) genes encoding transcription factors: at least some A-type CLEs can inhibit the expression of the *WUSCHEL* (*WUS*) gene in the shoot apical meristem and the *WOX5* gene in the root apical meristem [85,86]. At the same time, B-type CLEs activate the expression of *WOX4* in the procambium and cambium [87]. Members of the two different types of plant CLE peptides are likely to interact with each other at least in the control of cambium activity [88].

The first group of cyst nematode CLE genes encodes the A-type CLEs which belong to the same functional group as the AtCLE1-7 of Arabidopsis [77]. The overexpression of the genes from this group (e.g., HgCLE2 of H. glycines and GrCLEs of G. rostochiensis) can rescue the clv3 mutant phenotype in *A. thaliana* [50,55] indicating functional homology of nematode CLE peptides with plant A-type CLEs. The nematode-derived A-type CLE peptides can bind with plant CLE receptors in planta: for instance, HsCLE2 of beet cyst nematode *H. schachtii* was shown to bind to the CLAVATA1 (CLV1), CLAVATA2/ CORYNE (CLV2/CRN), BARELY ANY MERISTEM 1 and 2 (BAM1, BAM2), and RECEPTOR PROTEIN KINASE 2 (RPK2) receptors in *Arabidopsis* [51,52,53,57], and GrCLE peptides of *G. rostochiensis* are able to bind to the StCLV2 receptor in potato [56]. The genes encoding the CLV1, CLV2-CRN and RPK2 receptors are expressed in the syncytia induced by the *H. schachtii* in *Arabidopsis* roots [52], and the RNAi of the genes encoding these receptors in soybean was reported to enhance plant resistance to nematode *H. glycines* [54]. This suggests that the interaction of A-type CLEs secreted by cyst nematodes with plant receptors is necessary to promote nematode parasitism.

The second group of the CLE peptides from cyst nematodes, which is functionally similar to plant B-type CLEs, has been initially identified in *H. glycines* and *H. schachtii* [48]. Like plant B-type CLEs, nematode peptides of this group bind to the TDIF RECEPTOR (TDR) receptor, activate the expression of the *WOX4* gene, and stimulate both cambium activity and syncytium formation, since the expression of “cambium-related” *TDR* and *WOX4* genes were detected in nematode-induced syncytia [48]. In the same way as it was reported for *Arabidopsis* CLE peptides of A and B types [88], interactions of nematode A- and B-type CLEs have been identified. Exogenous treatment with both nematode A-type and B-type CLEs induces massive cell proliferation in wild-type roots, which suggests that both groups of CLE peptides may contribute to cell proliferation during feeding site formation [48].

In the reniform nematode *R. reniformis*, three *CLE* genes were identified. The proteins encoded by the *RrCLE* genes have the same structure as those of the cyst nematodes: they contain one C-terminal CLE domain (which is very similar to A-type CLEs of *Heterodera*), N-terminal signal domain, and a variable domain. The expression of *RrCLEs* was detected in the dorsal esophageal gland region of *R. reniformis* sedentary females [58].

The functions of CLE peptides of root-knot nematodes are less studied. The first identified CLE of the root-knot nematode was an *M. incognita* effector protein 16D10 with sequence similarity to the plant CLE peptides [60]. Further computational analysis of *Meloidogyne* spp. genomes revealed numerous candidate *CLE* loci in each species [61,89]; for example, the genome of *M. hapla* includes eight putative *16D10*-like genes [89]. Unlike CLEs of cyst nematodes, the 16D10-like CLE proteins of *Meloidogyne* spp. do not contain variable domain and include N-terminal secretion signal domain which is separated from the conserved C-terminal CLE domain by a predicted cleavage site [60]. The absence of the variable domains in the CLEs of *Meloidogyne* is consistent with their strategy of plant invasion. Unlike cyst nematodes, root-knot nematodes inject their glandular secretion containing peptide hormones directly into the apoplast, where they presumably interact with host receptors [89]. Based on sequence similarity of CLE domains, *M. hapla* and *M. incognita* have 16D10-like CLEs which are close to A- or B-type plant CLEs [89], but the effects of different types of *Meloidogyne* CLEs on plant development have not been studied. However, “cambium-associated” genes *WOX4* and *TDR* are activated in the feeding sites of both cyst- and root-knot nematodes [66,90], indicating closely related initiation mechanisms for both types of feeding site formation.

The overexpression of the *16D10* gene in *Arabidopsis* caused accelerated root growth [60], and the RNAi of *16D10* in potato increased the resistance of plants against five *Meloidogyne* species [59], which indicates the important role of these proteins in pathogenesis. A root developmental response in the *Arabidopsis* plants overexpressing *16D10* may be caused by the direct binding of the 16D10 proteins to the two host SCARECROW-LIKE proteins (AtSCL6/AtHAM3 and ASCL21), which was found in the yeast two-hybrid assay [60]. The SCLs are the members of the GRAS family of transcription factors, some of which play important roles in root tissues development and RAM (root apical meristem) specification [91,92]; however, the mechanism of 16D10 interaction with the nuclear-localized SCL proteins remains unclear.

Later, another group of the CLE-like proteins of root-knot nematodes was discovered: multiple CLE-like motifs were identified in the previously described MAP family of proteins, which were also found in nematode esophageal glands and which are secreted into the host plant tissues [62,63]. Unlike 16D10, the MAP proteins contain the variable domain with sequences similar to conserved sequences inside the variable domain of cyst nematode CLEs. Moreover, some of these domains are similar to conserved motifs from variable domains of *Heterodera’*s CLEs, whereas other domains show sequence similarity with such motifs from *Globodera*’s CLE proteins, and, therefore, they were named *Heterodera* VD-like motif (HVLM) and *Globodera* VD-like motif (GVLM), respectively [62]. The structure of MAP proteins, however, differs from that of cyst nematode CLEs; as a rule, MAPs contain several CLE domains, which can be located both at the C-terminus and in the middle part of the protein at a great distance from each other (in contrast to them, in multidomain CLEs of cyst nematodes, CLE domains are located in tandem at the C terminus). The GVLM and HVLM motifs can be located at the N- or C-terminal part of the MAP protein, as well as between the CLE-domains, and the same MAP protein may contain both GVLMs and HVLMs [62]. High level of sequence similarity of conserved sequences in the variable domains of MAPs and cyst nematode CLEs suggest that root-knot nematodes could use the same CLE-associated pathogenesis strategy as the one used by cyst nematodes. It is interesting that the MAP proteins are specifically present in “clonal” species of *Meloidogyne* that are reproduced by mitotic parthenogenesis, and have not been detected in the *Meloidogyne* species with other ways of reproduction [93]. However, the role of the MAP proteins in the development of nematode infection, as well as their effect on plant development, the possibility of processing in plant cells, and interaction with plant receptors have not been studied so far.

#### 2.1.2. CEP Peptides of Plant Parasitic Nematodes

The genes encoding the CEP proteins were firstly identified in the genome databases of three *Meloidogyne* species; at the same time, searches for the *CEP* genes in the genomes of cyst nematodes were unsuccessful [65]. Unlike plant CEP proteins, CEPs of *Meloidogyne* do not have a variable domain, and the N-terminal signal domain is directly adjacent to the C-terminal functional CEP domain. All 12 *CEP* genes of *M. hapla* are expressed in the tissues of nematode larvae prior to host invasion, which is consistent with their possible role in the host–parasite interaction [65]. The biological activity was demonstrated for the CEP2 peptide of *M. hapla* (MhCEP2): synthetic MhCEP2 inhibited primary root growth and lateral root formation similarly to MtCEP1.1 of *Medicago truncatula* and AtCEP5 of *Arabidopsis* [64].

More recently, syncytia-forming plant-parasitic nematode, *R. reniformis*, was also found to produce and secrete the CEP propeptides. The genome of this reniform nematode also contains 12 *CEP* genes, and some of them were shown to be highly upregulated during the infection phase of the nematode life cycle. Moreover, DIG-labeled *RrCEP1* transcripts were localized in the pharyngeal glands of juvenile and adult *R. reniformis* females suggesting that RrCEPs are secreted into the plant root. Unlike CEPs of root-knot nematodes, the RrCEP proteins contain a variable domain between the N-terminal signal domain and the C-terminal CEP domain. Moreover, some RrCEP proteins have several CEP domains in tandem. Since RrCEPs share no sequence similarity with plant CEPs and CEPs of *Meloidogyne* with the exception of the 15-amino-acid CEP domain, RrCEPs have been probably evolved independently of plant CEPs and root-knot nematode CEPs. At the same time, synthetic RrCEP1 demonstrated a similar, but less pronounced inhibitory effect on the primary root growth and lateral root formation as the plant peptides MtCEP1.1 from *M. truncatula* and AtCEP5 from *A. thaliana.* Moreover, when either RrCEP1.1 or AtCEP5 was supplemented to the growth medium, syncytia induced by *H. schachtii* were significantly smaller than those induced on the roots without peptide treatment, indicating the role of nematode and plant CEPs as negative regulators of feeding sites enlargement. This function of CEPs may allow nematodes to keep the size of feeding sites small to their own benefit, because over-sized feeding sites may drain excessive nutrients from the host plant and kill them [64]. In addition, synthetic RrCEP1, as well as the MtCEP1.1 and AtCEP5 peptides, increased the expression of the gene encoding *Arabidopsis* nitrate transporter (*AtNRT2.1*), which was previously found to be induced by plant CEPs. This suggests that RrCEP1, like plant-encoded CEPs, could regulate nitrate uptake in the host plant [64]. However, to date, nothing is known about the processing of nematode CEP propeptides in plants, as well as their interaction with plant receptors.

#### 2.1.3. IDA Peptides of Plant Parasitic Nematodes

Nematode-derived IDA peptides seem to be the effectors specific for root-knot nematodes: the *IDA*-like genes were identified in the genomic sequence of *M. incognita* and *M. hapla* [70], but no *IDA*-like genes were found in the sequence data for cyst nematodes [70,71]. *M. incognita* has two *IDA*-like genes, *MiIDL1* and *MiIDL2*, and the corresponding proteins contain N-terminal signal secretion domain and C-terminal conserved IDA domain, which are very similar to that of plant IDA peptides but do not contain a variable domain [70].

The MiIDL peptides can functionally mimic plant IDA peptides: the exogenous application of a synthetic MiIDL1, like synthetic IDA peptides of *Arabidopsis* and soybean, rescues the defects in petal abscission and root branching in the *Arabidopsis ida* mutant. The recovery of the delayed floral organ abscission phenotype was also observed when the full-length *MiIDL1* gene was introduced into the *ida* mutant [71]. At the same time, neither MiIDL1 nor native AtIDA peptide rescued petal abscission phenotype in the *hae*-3/*hsl2-3* double mutant with loss of function of both receptors for IDA peptides, indicating that MiIDL1 can be recognized by the HAESA (HAE) and HAESA-LIKE 2 (HSL2) receptors [71]. The expression of a nematode-targeted *MiIDL1*-RNAi construct in the host plant caused the formation of fewer and smaller galls in comparison with the control plants, suggesting that the nematode IDA peptides can play a role in the formation of galls during plant invasion by root-knot nematodes [70]. Taking into account the key role of the IDA peptides in cell wall dissolution leading to the separation of cell layer [37], it is assumed that the nematode-derived IDAs can play a role in the modification of the cell walls during the formation of feeding sites.

### 2.2. Peptide Phytohormones of Plant Pathogenic Fungi

Plants and fungi closely interact in the ecosystems. They often exist in a mutualistic symbiosis where fungi endophytically colonize plant roots increasing host plant resistance to stresses and enhancing nutrient acquisition and nutrient use efficiency. Saprotrophic fungi decompose plant debris. Finally, there are numerous species of plant pathogenic biotrophic and necrotrophic fungi. Pathogenic fungi cause the greatest harm to both natural and agricultural plant ecosystems: about 80% of yield losses due to phytopathogenic microorganisms are caused by fungal pathogens [94].

The plant pathogenic fungi can manipulate the development and immune response of the host plants via complex and diverse mechanisms, including the production of IAA and cytokinins [95], as well as the secretion of effector molecules, which mimic certain plant regulators to facilitate the infection [3]. Among such effectors, there are the homologs of plant peptide hormones [40].

To date, among phytopathogenic fungi, the effector peptides which mimic peptide phytohormones of the RALF, PSK, IDA, and PEP families have been found: the RALF and PSK peptides are quite common in the fungal kingdom and were identified in more than 20 Basidiomycota and Ascomicota species, while the IDA and PEP peptides have been revealed in only a few species [41,72,73].

#### 2.2.1. RALF Peptides of Plant Pathogenic Fungi

A fungal gene encoding a close homolog of plant RALF peptides was firstly identified in *Fusarium oxysporum,* a widespread pathogenic fungus with a broad host range. The RALF precursor protein encoded by *F. oxysporum* (F-RALF) lacks a variable domain and consists of the conserved RALF domain corresponding to a mature peptide, preceded by an N-terminal secretion signal domain [72]. The functional domain of F-RALF contains four highly conserved cysteine residues and the ‘ISY’ motif [72], which is required for the alkalinizing activity of plant RALF peptides [96].

Furthermore, the *RALF*-like genes were identified in the genomes of 26 species of biotrophic and necrotrophic phytopathogenic fungi, which belong to the Pucciniomycetes class of the Basidiomycota and the Dothideomycetes and Sordariomycetes classes of the Ascomycota; some of these fungi species have even several *RALF*-like genes [72,73]. All of the fungi species which were found to possess the *RALF* genes are plant pathogens, indicating the role of RALFs in fungal pathogenesis [73]. Most of the identified fungal *RALF* genes have sequence similarity to *Arabidopsis AtRALF1* or *AtRALF27.* The fungal *RALF* gene sequences can vary quite a lot within a species: for example, different subspecies and isolates of *F. oxysporum* differ in the sequences of their *RALFs* [73].

The RALF peptides of *F. oxysporum* f. sp. *lycopersici* demonstrated the biological activity which is similar to that of the plant RALF peptides. In different experiments, two from three analyzed synthetic F-RALF peptides corresponding to RALFs of *F. oxysporum* f. sp. *lycopersici* from different isolates, caused the inhibition of root elongation and root hair growth in tomato and *Arabidopsis* seedlings in the same way as the tomato SlRALF peptide [72,73]. At the same time, F-RALFs, like plant RALFs, triggered a rapid and concentration-dependent extracellular alkalinization of tomato suspensions culture, as well as reactive oxygen species burst and activation of MAP-kinases in the leaf explants of tomato and *Nicotiana benthamiana* [72,73]. Moreover, the F-RALF-dependent root growth inhibition was shown to depend on the alkalinization rate: it could be partially reversed by lowering the extracellular pH, whereas the increase in pH stimulated the root growth-inhibitory effect even in the absence of F-RALF [72].

The expression of the *F-RALF* gene in *F. oxysporum* f. sp. *lycopersici* is dramatically increased during the infection of tomato, which indicates the role of F-RALF in *Fusarium* pathogenesis [72,73]. This role could depend on alkalinization effect of F-RALFs: mutant strains of *F. oxysporum* f. sp. *lycopersici* with loss of *F-RALF* gene function were unable to cause alkalinization of extracellular space and reactive oxygen species burst during the infection of tomato plants, but the capacity to alkalinization was fully restored in the complemented strains [72]. Moreover, mutants of *F. oxysporum* f. sp. *lycopersici* lacking F-RALF showed a reduced capacity to colonize the tomato plants, and this capacity was restored in the complemented strains [72].

The efficiency of *F. oxysporum* in the invasion of host plants depends on the activity of the RALF receptor, LRR-RLK FER [97]. The *Arabidopsis* mutant *fer-4,* which lacks a functional copy of the *FER* gene, did not arrest root elongation in response to F-RALF, as well as in response to inoculation with *F. oxysporum* [72,73].

At the same time, in the other *Fusarium* species, *F. graminearum*, the F-RALF seems to be unnecessary for its virulence. The mutant strains lacking their single *RALF* gene, *FgRALF*, exhibit normal virulence to wheat and *Arabidopsis* floral tissue, and transient virus-mediated overexpression of *FgRALF* in wheat only slightly increased the rate of colonization of floral tissue by *F. graminearum* [74]. Thus, the conserved role of fungal RALFs in the pathogenesis of different fungal pathogens remains to be questionable.

#### 2.2.2. PSK, IDA and PEP Peptides of Plant Pathogenic Fungi

In addition to RALFs, phytopathogenic fungi can also synthesize peptide phytohormones of other groups. Analysis of sequenced genomes of numerous plant pathogenic fungi revealed the genes, which presumably encode the precursors of the PSK, IDA, and PEP peptides. The *PSK* genes are quite widespread in the fungal kingdom, whereas the *IDA* and *PEP* genes were found in only a few species of fungi [41].

To date, more than 20 fungi species from Basidiomycota (Tilletiaceae family) and Ascomicota (Glomerellaceae, Botryosphaeriaceae and Mycosphaerellaceae families) were identified as the ones containing the homologs of plant PSK peptides. Most of these fungi species have a single *PSK* gene, however, *Colletotrichum higginsianum* (Ascomicota), which is a causative agent of economically important anthracnose diseases on numerous monocot and dicot crops [98], contains three *PSK* genes [41]. Fungal PSK homologs are divided into two divergent groups [41]. The PSK proteins from five species of the genus *Tilletia* (Basidiomycota), which infect cereals [99], contain N-terminal signal domain and C-terminal conserved PSK domain [41], whereas PSKs of different Ascomycota species contain a single signal domain and multiple (from two to eight) repeated PSK domains [41].

IDA homologs were identified in the two species of phytopathogenic fungi [41]: *Melampsora larici-populina* (Basidiomycota), which is the main rust pathogen of different species of *Populus* [100], and *Colletotrichum fructicola* (Ascomicota), a pathogen with a broad range of host plant species [101]. The IDA protein of *M. larici-populina* contains a conserved C-terminal IDA domain, but lacks an N-terminal signal domain, whereas the IDA of *C. fructicola* contains both predicted N-terminal signal and C-terminal functional domains [41].

Finally, one genus of epiphyte and conditional pathogenic fungi, ubiquitous non-*Saccharomyces* yeast *Metschnikowia* (Ascomicota), has a gene encoding the homolog of plant PEP peptides. The PEP protein of *Metschnikowia* contains a typical C-terminal PEP domain and its sequence was clustered with the *Arabidopsis* PEP5 protein [41].

Thus, phytopathogenic fungi appear to be capable of synthesizing four different families of peptide phytohormones. In some species of fungi, homologs of plant genes encoding peptides of two different families have been found: for example, *C. fructicola* has both an *IDA-*like gene and a *PSK-*like gene. At least for one of the *Fusarium* species, *F. oxysporum*, an important role of the fungal RALF peptide in plant colonization was shown [72,73]. At the same time, all other fungal homologs of peptide phytohormones have been identified quite recently [41], and their roles and mechanisms of action remain unknown.

### 2.3. Peptide Phytohormones of Plant Pathogenic Bacteria

Currently, over 300 species of bacteria are known to cause diseases in various plants [102]. Bacterial plant diseases cause enormous economic damage, affecting valuable species of agricultural plants. Phytopathogenic bacteria colonize either a plant surface or its tissues and cause various symptoms such as wilting, spots, blights, cankers, rots, tissue overgrowth, stunting, root branching, leaf epinasty, etc. [102]. The ability of plant-associated bacteria, both pathogenic and beneficial, to produce plant growth regulators has long been known [14,19,20]. Recently, it was shown that some phytopathogenic bacteria can also produce the effectors that mimic peptide phytohormones. In the genomes of some species of plant-interacting bacteria, the homologs of plant genes encoding peptide phytohormones of the CLE, CEP, RALF, PSK, PSY, and PEP families have been identified [41,66,67,73]. Some of these bacteria, namely *Xanthomonas* species which produce PSY-like proteins [66,67], and also two bacterial species possessing RALF-like and CEP-like sequences [41], correspondingly, are plant pathogens (see below).

#### 2.3.1. PSY Peptides of Plant Pathogenic Bacteria

The most studied example of bacterial effectors which mimic peptide phytohormones is a PSY-like protein named RaxX [67] produced by hemibiotrophic bacterium *Xanthomonas oryzae* pv. *oryzae*, a causative agent of leaf blight disease, which was included in the top 10 most economically significant phytopathogenic bacteria [103]. The study of RaxX began with the discovery of the rice *Xa21* gene, which encodes an LRR-RLK and which confers plant resistance to *X. oryzae* pv. *oryzae* [104]. Searching for the genes of *X. oryzae*, that are required for the activation of Xa21 (*Rax* genes) led to the identification of the gene named *RaxX*, encoding a small protein with a tyrosine sulphation site (Y41) and a predicted N-terminal signal domain, which shares similarity to plant peptides of the PSY family [66,67]. The *RaxX* genes were found in many *Xanthomonas* species, and a highly conserved *RaxX* sequence across species indicated its important biological function [66,68].

The RaxX protein was shown to be sulphated at the Y41 site by the prokaryotic tyrosine sulphotransferase RaxST, which is required for the activation of Xa21. The infection assays using bacterial mutants that lack *RaxX*, or carry the mutations in the Y41, demonstrated that such mutant strains were impaired in virulence and the expression of defense-related genes [66]. Therefore, the RaxX peptide, especially its Y41 residue, is required for the activation of Xa21-mediated plant immunity. Moreover, the full-length sulfated recombinant RaxX (but not its non-sulphated variant) triggered defense gene expression in the leaves of rice plants overexpressing *Xa21*. The test of immune activity of chemically synthesized N- and C-terminal parts of RaxX protein showed that the tyrosine-sulfated C-terminal part of RaxX is sufficient to activate Xa21-mediated defense responses [66,67].

The product of another Xa21-activating gene of *X. oryzae*, encoding a bifunctional protease/transporter RaxB, is necessary for the maturation of the RaxX precursor protein into a 16 amino acid peptide. Therefore, RaxX is secreted from *Xanthomonas* as a proteolytically processed and sulfated mature peptide. In the N-terminal domain of the RaxX precursor protein, the residues that are critical for the RaxX peptide maturation and secretion have been identified [69]. Thus, bacterial pathogen *X. oryzae* pv. *oryzae* ensures RaxX maturation by itself and secretes a mature peptide [69]. This is in contrast to phytopathogenic fungi and nematodes which secret the precursors of peptide phytohormones and which are suggested to use host plant machinery for their maturation [56,72].

It was also shown that synthetic sulfated RaxX derivative comprising 13 amino acid residues, which are highly conserved between RaxX and plant PSY peptides, induces root growth in *Arabidopsis* and rice mimicking the root growth-promoting activities of the *Arabidopsis* AtPSY1 peptide. The 13 amino acid RaxX from diverse *Xanthomonas* species also demonstrated AtPSY1-like activity in the promotion of root growth in *Arabidopsis* seedlings, indicating the conserved function of this protein in *Xanthomonas.* At the same time, the AtPSY1 peptide, unlike RaxX, was unable to activate Xa21-mediated immunity in rice plants [66].

Finally, it was shown that the mature sulphated RaxX peptide, but not *Arabidopsis* AtPSY1, directly and with high affinity binds to Xa21 LRR-RLK of rice [69]. Therefore, this PSY-like protein of phytopathogenic bacterium *X. oryzae* pv. *oryzae* specifically interacts with a plant receptor to facilitate the infection.

#### 2.3.2. RALF and CEP Peptides of Plant Pathogenic Bacteria

Nine species of Actinobacteria, most of which are plant pathogenic, e.g., *Streptomyces acidiscabies* causing potato scab disease, possess putative secreted proteins containing a C-terminal RALF peptide domain. The peculiarity of the bacterial RALF proteins is a domain homologous to the S1 pertussis toxin subunit, which is absent in plant and fungal RALFs [73]. However, no biological function has been reported for these peptides.

Bacterial CEP homolog was revealed in *Ralstonia syzygii* (Proteobacteria), a pathogen causing bacterial wilt disease on a wide range of host plants [105]. This bacterial CEP was grouped together with the *Arabidopsis* CEP5 protein but its functions have not yet been studied [41].

## 3. Peptide Phytohormones from Plant Symbiotic and Beneficial Microbes: *Si vis pacem, para bellum*

In addition to plant pathogens, symbiotic and beneficial microbes are also able to alter plant developmental processes by producing phytohormones or by changing their balance in the host plant. Since beneficial microbes are initially recognized as potential invaders, molecular interference with the plant immune system, including “defense-related” phytohormones, is fundamental for their survival and the establishment of a mutualistic relationship with the host plant [106].

JA was shown to be accumulated in the host plant roots at the early stages of infection by mycorrhizal fungi as well as rhizobia [11]. In the rhizobia, JA can induce the production of Nod factors—the lipochitooligosaccharide signals, which are secreted into the rhizosphere and which are required for nodule development [107]. The Nod factors, in its turn, were shown to suppress plant immune response to both rhizobia and other pathogenic bacteria [108]. On the other hand, genes encoding JA biosynthetic enzymes and the MYC2 transcription factor, which is involved in JA signaling, are negatively regulated by the GmNARK (Glycine max Nodule Autoregulation Receptor Kinase) receptor kinase, an LRR-RLK which is activated during symbiosis with rhizobia and plays a central role in the regulation of nodule number [109].

The mycorrhizal fungi can modulate ethylene signaling to possess an immune-suppressive function. Thus, during plant colonization with arbuscular mycorrhizal fungus *Glomus intraradice*, fungal effector protein SP7 directly interacts with the pathogenesis-related Ethylene Response Factor 19 (ERF19) transcription factor, which is often induced by ethylene and fungal pathogens to overcome the ethylene-dependent plant defense system [15]. Other plant growth-promoting bacteria and fungi can also modulate plant defense via SA-JA antagonism or suppression of ethylene signaling. For instance, rhizosphere bacteria *Rhizobacteria* spp. possess ACC deaminase, an enzyme that degrades the ethylene precursor ACC and thus decreases the ethylene level in the host plant [16].

However, there are only a few examples of peptide phytohormone mimicry in the plant symbiotic and beneficial microbes.

### 3.1. Possible Homologues of Peptide Phytohormones in Plant Beneficial and Plant-Associated Non-Pathogenic Bacteria

The most well-known example of beneficial plant-microbe interaction is the formation of the nitrogen-fixing symbiosis of legume plants with soil bacteria collectively known as rhizobia. In response to a signaling cascade triggered by Nod-factors, lipochitooligosaccharidic molecules produced by rhizobia, the activation of pericycle and cortex cell divisions in the root of the host plant leads to the formation of nodules, where rhizobia are differentiated into bacteroids to fix atmospheric nitrogen for the host plant benefit [110]. Cytokinins are the key hormones that stimulate root cell divisions upon nodulation [111], and the increase in plant cytokinin biosynthesis gene expression is observed in the sites of rhizobia inoculation [112]. It was found that rhizobia themselves secrete cytokinins and IAA [113]. However, bacterially produced cytokinins were found to be insufficient to allow nodulation [114], and IAA secreted by rhizobia does not influence nodulation efficiency [115]. The ability to produce IAA makes rhizobia important regulators of plant root system development and allows to consider them as plant growth-promoting rhizobacteria (PGPR), which could increase the yield of non-leguminous species [116]. However, the genes encoding for the possible precursors of peptide phytohormones have not yet been described in rhizobia.

At the same time, in other bacterial species of Actinobacteria, Proteobacteria, and Gemmatimonadetes phyla, however, the genes encoding the possible homologs of the CLE, CEP, PSK, and PEP peptides have been identified [41]. Among them, *Actinobacteria* sp., which are considered as PGPR [117], encode two putative homologs of plant *CLE* peptides. The products of these genes lack a putative signal peptide sequence [41]. Interestingly, the CLE peptide motif of one of them (HBW17759.1) has high similarity with the plant nitrate-regulated CLE peptides, including the AtCLE1-7 peptides from *A. thaliana* and legume CLE peptides which are known as negative regulators of the symbiotic nodulation [118]. It is of great interest to study if the identified CLE peptide-encoding gene from *Actinobacteria* sp. could affect plant root growth and plant interaction with rhizobia. The hypothetical protein encoded by a single *CLE* gene of uncharacterized soil bacterium *Gemmatimonadetes* sp. (Gemmatimonadetes) is closer to the AtCLE1, AtCLE3, and AtCLE4 peptides, as well as to CLEs of cyst nematodes, and contains an N-terminal signal domain, whereas CLE of *Thiotrichales* sp. (Proteobacteria) is closer to AtCLE19 and AtCLE21 and lacks a signal domain [41].

In addition, one PSK and one PEP homologs have been identified recently in two plant-associated non-pathogenic bacteria species, but their functions have not yet been studied. The PEP-like protein of *Mycolicibacterium conceptionense* (Actinobacteria) was clustered with the *Arabidopsis* PEP7 protein. The PSK homolog containing the C-terminal PSK domain and N-terminal signal domain was identified in *Proteobacteria* sp. isolated from phyllosphere metagenome [41].

### 3.2. The Homologues of Plant Peptide Phytohormones in Arbuscular Mycorrhizal Fungi

Another example of beneficial plant–microbe interaction is plant symbiosis with arbuscular mycorrhizal fungi (AMF), which occurs in 85% of the vascular plant species [119]. AMF colonizes plant roots and helps the host plant to efficiently absorb minerals, especially phosphate, from the soil. The establishment of this type of symbiosis involves signaling exchange between the symbiotic partners, and AMF was shown to produce bioactive plant hormones such as cytokinin (isopentenyl adenosine) and IAA [120].

Moreover, in the genomes of AMF fungi (four *Rhizophagus* species and one *Gigaspora* species), the genes encoding CLE-like peptides have been identified [42]. These genes encode precursor proteins with an N-terminal signal domain for secretion and the C-terminally located conserved CLE domain. The sequences of the AMF-encoded CLE peptides have high similarity with certain A-type CLE peptides, namely AtCLE14 of *A. thaliana* and MtCLE5 of *M. truncatula*. For the AtCLE14 peptide, the role in root growth regulation under low phosphate conditions was reported [44], whereas MtCLE5 was shown to regulate root architecture in *M. truncatula* by inhibiting primary root elongation and stimulating lateral root formation [45]. Therefore, it was suggested that the AMF-encoded CLE peptides could mimic native plant CLEs to regulate root growth under plant colonization by mycorrhizal fungi [42].

For two AMF species, *Rhizophagus irregularis* and *Gigaspora rosea*, the activation of the *CLE* genes (*RiCLE1* and *GrCLE1*) under AMF symbiosis development was shown. Moreover, synthetic RiCLE1 peptide treatment affected root system development in *M. truncatula*: it inhibited the growth of primary roots and stimulated the formation of lateral roots. This effect was less severe in the *clv2* mutants both in pea and *A. thaliana*, suggesting that the CLV2 protein could be responsible for the perception of RiCLE1. In addition, exogenous application of RiCLE1 to the seedlings prior to inoculation with *R. irregularis* enhanced further mycorrhization of the root [42]. These data strongly suggest that the AMF-encoded CLE-like peptides may modulate root growth and positively regulate the establishment of arbuscular mycorrhizal symbiosis [42].

Various plant-interacting bacteria, fungi, and nematodes have the genes encoding the precursors of plant peptide phytohormones of CLAVATA3/EMBRYO SURROUNDING REGION-RELATED (CLE), PLANT PEPTIDES CONTAINING SULFATED TYROSINE (PSY), phytosulphokines (PSK), C-TERMINALLY ENCODED PEPTIDES (CEP), INFLORESCENCE DEFICIENT IN ABSCISSION (IDA), RAPID ALKALINIZATION FACTOR (RALF), and PLANT ELICITOR PEPTIDES (PEP) families. All these proteins contain one or several conserved functional domains, which are essential for peptide function, and most of them also have a signal domain which is necessary for secretion, and/or variable domain. Several examples of such non-plant peptide phytohormone precursors, such as the RALF protein of *Fusarium oxysporum,* the CLE, CEP, and IDA proteins of plant-parasitic nematodes, and the CLE proteins of arbuscular mycorrhizal fungi, can undergo processing in plant cells to produce short mature peptides. These peptides can bind with the receptors for corresponding families of plant peptide phytohormones. In contrast, PSY-like RaxX protein of *Xanthomonas oryzae* pv. oryzae undergoes processing in the bacterium and binds to the specific plant receptor for RaxX, but not to the receptors of the plant PSY peptides.

## 4. Discussion: Strategy of Information War and “Spy Games” with Peptide Phytohormones

It is well known that some plant pathogens and beneficial plant-interacting organisms are able to use phytohormones to increase the efficiency of host plant colonization. Since phytohormones coordinate plant growth in response to environmental and developmental stimuli, pathogens with the ability to manipulate phytohormonal level and/or response could misinform the host plant about the current status of the external and internal environment, forcing it to change the developmental strategy of the plant organism. Therefore, such pathogens, in addition to the usual “race of arms”, can use the strategy of “information war” against the host plant. According to the definition of military experts, the main properties of an information war are: a flexible arsenal of weapons and high unpredictability; gradual conquest of territories; imperceptible impact on the enemy, which can be clothed in a benevolent form; lack of visible destruction, as a result of which the defense mechanisms of society are not activated. Indeed, these methods can be used by phytohormone-manipulating pathogens to invade a host plant.

Various plant pathogens and symbionts can produce effector proteins that mimic peptide phytohormones, and some of such effectors were shown to bind with plant receptors that perceive the corresponding phytohormornes of plant origin and trigger downstream signaling pathways [53,56,57,69,72,73]. In turn, plants can also use their systems of perception and signaling of peptide phytohormones to limit the colonization level by pathogens and symbionts. For example, biosynthesis of certain plant CLE peptides and activation of their receptors upon plant colonization with rhizobia form the basis of the system, named Autoregulation of Nodulation (AON), which limits the number of nodules per plant [121]. The CLE signaling pathways, including those participating in AON, were also shown to regulate the colonization of legume plants with arbuscular mycorrhizal fungi [122]. Moreover, according to our data, the AON system can be induced under the colonization of legume plants with bacterial pathogen *A. tumefaciens* [123]. Interestingly, the transcriptomic analysis of developing syncytia in soybean roots under invasion by nematode *R. reniformis* revealed the upregulation of numerous genes which had previously been associated with rhizobia nodulation, such as nodule-initiating transcription factors CYCLOPS, NSP1, NSP2, and NIN, as well as multiple nodulins associated with the plant-derived peribacteroid membrane [80]. A possible similarity of the regulatory mechanisms underlying nodulation and development of nematode galls on roots was suggested in earlier studies that showed that plants carrying mutations in the *HAR1* locus in *L. japonicus* [124] are hyper-infected by root-knot nematodes [125]. Thus, plants can use conserved regulatory systems, including those involving the signaling of peptide phytohormones, to interact with various pathogens and symbionts.

Most likely, the ability to synthesize effectors which mimic peptide phytohormones evolved independently in different groups of plant-interacting organisms. An important question is how the genes of peptide phytohormones appeared in plant parasites and symbionts: whether it happened due to long-time coevolution of the parasite/symbiont and the host or as a result of horizontal gene transfer (HGT). On the one hand, the coevolution hypothesis seems to be more logical than HGT, since the strong evidence supporting the HGT hypothesis (for instance, phylogenetic incongruence between species and gene trees) is lacking. On the other hand, relatively short sequences of the genes encoding peptide phytohormones have a restricted phylogenetic signal, making it difficult to obtain data on the phylogeny of such peptides [89]. In addition, genes encoding non-plant peptide phytohormones are characterized by a high degree of similarity with plant peptide phytohormone precursors in the sequences of the functional domains (CLE, CEP, etc.), and N-terminal signal domain, but, as a rule, do not show any sequence similarity with the plant sequences outside the domains which are important for peptide maturation, secretion and further functioning [41]. Moreover, the HGT of “phytohormonal” genes from plants to fungal pathogens/ symbionts or parasitic nematodes seems to be unlikely since HGT between eukaryotes was considered to be a quite rare evolutionary event [126]. However, numerous cases of HGT from prokaryotes to eukaryotes have now been reported [127]. Thus, although it cannot be ruled out that these gene families encoding peptide phytohormones were descended from a common ancestor, it is usually assumed that non-plant peptide phytohormones may have arisen de novo (convergently) [89].

Finally, the hypothesis of the coevolutionary origin of non-plant peptide phytohormones gained popularity in connection with such an origin of other phytohormones of non-plant origin, such as IAA and cytokinins. The host–parasite coevolution unambiguously explains a number of colonizing strategies of various phytopathogens and plant-beneficial organisms such as the ability to influence plant growth via pathogen/symbiont-derived IAA and cytokinins. Auxins and cytokinins are evolutionally older than plants and are present in many organisms, including bacteria, amoebae, filamentous fungi, nematodes, arthropods, etc. [19,20]. For example, it is assumed that over 80% of the rhizosphere bacteria are able to synthesize IAA [120]. The evolutionally conserved function of cytokinins in a broad range of organisms consists in their role as a component of tRNA which serve mainly in improving the translation efficiency and fidelity [128]. At the same time, IAA produced by bacteria and fungi was considered to be a secondary metabolite resulting from a detoxification process when tryptophan starts to accumulate in the cells, and, in addition, IAA is used as a signal for gene regulation in some bacteria [19]. In plants and various plant-interacting organisms, IAA and cytokinins acquired more specific roles as growth regulators. Therefore, plant pathogens and symbionts, which had the enzymes involved in IAA and cytokinin biosynthesis and modifications, gained an advantage in plant colonization [19,20].

In the case of non-plant peptide phytohormones, the coevolution, most likely, took place just for the most promoted case of peptide-phytohormonal mimicry in bacteria—namely, the PSY1-like RaxX peptides of *Xanthomonas* species [66,68], and ample evidence supports this hypothesis. First, the bacterium itself is responsible for the maturation of the RaxX peptide, including proteolytic processing and tyrosine sulfation, because it has all the necessary enzymes to do this [69]. To date, *Xanthomonas* is the only known plant pathogen that secretes a ready-made peptide phytohormone. Other plant-interacting organisms which are able to mimic peptide phytohormones do not have their own maturation enzymes and probably use the host plant machinery to produce mature peptides, as was demonstrated for the CLE peptides of cyst nematodes [56]. Second, the RaxX peptide specifically interacts with the plant Xa21 receptor providing a plant growth response similar to plant PSY1 peptide, but Xa21 does not bind to plant PSY1 [66,69]. Therefore, the Xa21 protein is not a plant hormone receptor per se (unlike many other receptors of peptide phytohormones which are able to bind plant peptides as well as non-plant ones), but rather a specialized receptor for a bacterial effector. That is, the RaxX-Xa21 system, in our opinion, represents a typical case of gene–gene interaction and the part of the effector-triggered plant immune system which includes highly specific plant LRR-RLKs or intracellular LRR-containing immune receptors and which has been evolved as a result of long-term co-evolution of pathogens and their hosts [1]. However, *Xanthomonas* is able to “deceive” the plant immunity since the capture of the RaxX effector by the Xa21 receptor facilitates the infection [66,67]. At the same time, there are many other examples of bacterial and fungal pathogens which also can hijack the plant immunity by producing an effector molecule that binds to the immune receptors to provide disease susceptibility [1].

Although RaxX most probably has evolved as a result of plant–pathogen coevolution, the emergence of other non-plant peptide phytohormones due to coevolution or HGT remains to be controversial, since HGT between plants and plant-interacting bacteria is not a rare event [129,130]. Among the effectors produced by phytopathogens, there are definitely those that arose as a result of HGT, for example, the cell wall degrading enzymes of plant-parasitic nematodes. A series of plant cell wall degrading enzymes, including cellulases, xylanase, pectate lyases, polygalacturonases, and also expansin-like proteins were identified in a variety of plant-parasitic nematode species [131]. All these enzymes showed a high level of similarity to the corresponding bacterial proteins: for example, the closest relatives of polygalacturonases GH28 of *Melioidogyne* species are found in *Ralstonia solanacearum*, a plant-pathogenic soil bacterium that shares plant hosts with these nematodes, while their PL3 pectate lyase clusters with corresponding enzymes of another plant-pathogenic bacterium, *Clavibacter michiganensis* [132]. The reduced virulence of nematodes after knock-down of the genes encoding cell wall degrading enzymes highlights their role in the successful infection. At the same time, non-plant parasitic nematodes normally do not need these enzymes for their life cycle and such genes have not been identified in their genomes [131]. Examples of HGT-originated genes in plant-parasitic nematodes are not limited by the genes for cell wall degrading enzymes: in general, the broad phylogenetic analysis provided evidence that about 3.5% of genes in these nematodes have a non-metazoan origin and might have been acquired from bacteria or fungi [131,133].

Since the examples of HGT of genes encoding effector proteins from bacteria to nematodes are not so rare, we can speculate that genes encoding non-plant peptide phytohormones might also have been acquired by eukaryotic pathogens from the bacterial intermediate. Indeed, quite recently, the CLE-, RALF-, CEP-, PSK-, and PEP-like genes have been found in the genomes of phytopathogenic and epiphytic bacteria, as well as PGPR, but they have not been studied at all up to date [41]. We also can assume that in the case of non-plant peptide phytohormones found in eukaryotes, bacteria might have acted as mediators in the transfer of genes from plants to other eukaryotes, since numerous examples of HGT from plants to bacteria and from bacteria to various eukaryotes have been described [126]. Indeed, some bacterial genes for peptide phytohormones were shown to cluster with well-defined plant peptides: for example, CLEs from Proteobacteria are close to AtCLE19, AtCLE20, and AtCLE21, while CLEs from Gemmatimonadetes and some Actinobacteria, like most cyst nematode CLEs, are close to AtCLE1/3/4 [41].

The origin of the genes encoding non-plant peptide phytohormones via co-evolution or HGT indicates that a molecular dialog of plants with their “enemies” and “allies” has been preceded by “spy copying of the military technologies” (in case of co-evolution) and even “theft of ready-made samples”, which might have happened directly or through bacterial intermediaries (in the case of HGT). This is not unique to plants: the acquisition of genes that facilitate interaction with the host through HGT has been observed in the pathogens of animals and other animal-interacting organisms. For example, in the genomes of necrophagous nematodes of the *Pristionchus* genus which feed on the remains of dead insects along with their microsymbionts, both cellulase genes from the bacterial donor, and the *Diapausin* genes, which encode antifungal peptides specifically produced during diapause, from insect donor, were found [134]. Therefore, the arms race and all sorts of spy life hacks that can be found in human society, have not been invented by humans: all these “techniques” have existed in nature for millions of years before them.

## 5. Concluding Remarks

Peptide hormones produced by plant pathogens, symbionts, and microbes interacting with plants are part of the molecular interface that ensures their coexistence with the host plant. The effectors produced by such organisms that mimic peptide phytohormones, as well as other effectors of pathogens and symbionts, are received by plant receptors, regulating not so much plant defense responses as growth responses. The origin of non-plant peptide phytohormones is controversial. Different peptides could arise either as a result of the coevolution of a pathogen/symbiont and a host plant, or as a result of horizontal gene transfer, and in our review, we discuss both these possibilities. In recent years, a lot of data have been accumulated both on the functions of plant peptide hormones and on the identified homologs of peptide phytohormones outside the plant kingdom. New discoveries in this area should help to resolve the issue of the origin of these unique peptides.

## Figures and Tables

**Table 1:** Peptide phytohormones from plant-interacting bacteria, fungi, and nematodes: protein structure, functional domain sequences, and plant receptors.

Family of Peptide Phytohormones	Species Name	Examples of Functional Domain Sequences	Domain Structure of Precursor Protein *	Putative Plant Receptor	References
CLE	**Bacterial CLEs**				
	*Actinobacteria* sp.	RLSPGGPDPARH RTIPTGSNPLRN	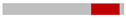	Unknown	[41]
	*Thiotrichales* sp. *Acidimicrobiaceae* sp.	RGIPTGPDPTHN			
	*Gemmatimonadetes* sp. *Actinobacteria* sp.	RVVPGGPRPVHH RVVPGGPRPIYH	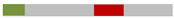		
	**Fungal CLEs**				
	*Rhizophagus irregularis*	RTVPSGPNPLHN RIVPSGPNPLHN	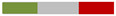	CLV2	[42,44,45]
	*Rhizophagus diaphanus*	RLVPSGPNPLHN			
	*Rhizophagus cerebriforme*	RIVPSGPNPLHN			
	*Rhizophagus clarus*	RTVPTGSNPLHN			
	**Nematode CLEs**				
	Cyst nematodes:				
	*Heterodera* spp.	RLSPSGPDPHHH RVSPSGPDPQHH RLSPSGPDPRHH HEVPSGPNPTQN	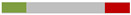	CLV1, CLV2/CRN, RPK1, BAM1, BAM2, TDR	[46,47,48,49,50,51,52,53,54]
	*Globodera* spp.	RVTPGGPDPLHN RVTPGVPDRQHR RVAGAGPDPIHN RAVPAGPDPKHN RGAPAGPDPIHN RVVVGGPDPQHH	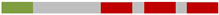 from 1 to 5 CLE domains	CLV2	[55,56,57]
	Reniform nematodes:				
	*Rotylenchulus* spp.	RESPGGPDPKHH	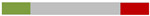	Unknown	[58]
	Root-knot nematodes:				
	*Meloidogyne* spp. 16D10	GKKPSGPNPGGN	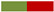	Unknown	[59,60,61]
	*Meloidogyne* spp. MAP	NVYPSGPEPSTP NLYPSGPEPTPK NPYQSGPEPSTQ NPYPSGPEPTPK NVYPSGPESSTP GKYPSGPPSHNQ	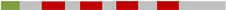 from 1 to 9 CLE domains	Unknown	[62,63]
CEP	**Bacterial CEPs**				
	*Ralstonia syzygii*	DSQPTTPGHSPGVGH	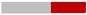	Unknown	[41]
	**Nematode CEPs**				
	Reniform nematodes:				
	*Rotylenchulus* spp.	AFKPTTPGHSPGAGH AFKPTTPGHSPGDGH GFRPTTPGHSPGVGN GFRPTTPGHAPGFGN AFRPTTPVHSPGAGQ PFKPTAPGHSPGVGH	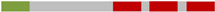 from 1 to 9 CLE domains	Unknown	[64]
	Root-knot nematodes:				
	*Meloidogyne* spp.	DPRPTNPGHSPGIGH AFRPTAPGHSPGVGH GYQPTNPGHSPGIGH PFKTVPGQSSPGVGH VIKPACIGNSPGVGH AFRPTNPGPSPAIGN	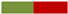	Unknown	[65]
PSY	**Bacterial PSY**				
	*Xanthomonas oryzae* pv. *oryzae* other *Xanthomonas* species	DYPPPGANPKHDP	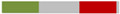	Xa21	[66,67,68,69]
PSK	**Fungal PSKs**				
	*Tilletia* sp.	YIYTQ	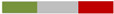	Unknown	[41]
	*Colletotrichum* sp. *Lasiodiplodia* sp. *Diplodia* sp. *Macrophomina phaseolina* *Cercospora* sp. *Ramularia collo-cygni* *Pseudocercospora* sp. *Zymoseptoria* sp.	YIYTQ	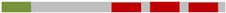 from 2 to 4 PSK domains		
	**Bacterial PSKs**				
	*Proteobacteria* sp.	YIYTQ	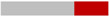	Unknown	[41]
IDA	**Fungal IDAs**				
	*Melampsora larici-populina*	PIPPSTTSKRHA	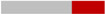	Unknown	[41]
	*Colletotrichum fructicola*	KIPPSGPSKKHN	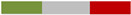		
	**Nematode IDAs**				
	*Meloidogyne* spp.	KNIPVPASGPSKRHN RGEPIPGSGPSVRN KGVPPNSGPSRRGN KGVPHNSAPSRRGN KGNKKSSGPSHRGN KGKKESSGPSLGGN	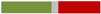	HAE, HSL2	[70,71]
RALF	**Fungal RALFs**				
	*Fusarium oxysporum* other 26 fungi species	..ISY…-[PCS…….NCR]- -[RGC…CRG]-	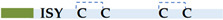 ISY—‘ISY’ motif, C—cysteine residues predicted to form disulfide bonds	FER	[72,73,74]
	**Bacterial RALFs**				
	*Streptomyces acidiscabies* other 8 species of Actinobacteria	..ISY…-[PCS…….NCR]- -[RGC…CRG]-	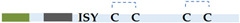 ISY—‘ISY’ motif, C—cysteine residues predicted to form disulfide bonds	Unknown	[73]
PEP	**Fungal PEPs**				
	*Metschnikowia* sp. JCM 33374	SSGIGGADN	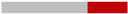		[41]
	**Bacterial PEPs**				
	*Mycolicibacterium conceptionense*	SSGTSGGSN	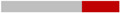	Unknown	[41]

* Domain structure of precursor protein: the red box corresponds to a functional domain, the green box—to a signal domain, the gray box—to a variable domain, the black box—to the S1 pertussis toxin subunit of bacterial RALFs.

## Data Availability

Not applicable.
